# Technology-based interventions to promote physical activity in Gulf Cooperation Council (GCC) countries: a scoping review protocol

**DOI:** 10.1136/bmjopen-2025-110795

**Published:** 2026-07-20

**Authors:** Asma Saad A Alghamdi, Marco Bardus, Manami Das, Khalifa Elmusharaf

**Affiliations:** 1 Applied Health Sciences, University of Birmingham, Birmingham, UK; 2 University of Birmingham - Edgbaston Campus, Birmingham, UK; 3 Centre for National Training and Research Excellence in Understanding Behaviour -CENTRE-UB, University of Birmingham, Birmingham, UK; 4 University of Birmingham, Birmingham, UK; 5 Department of Applied Health Sciences, University of Birmingham Dubai, Dubai, UAE

**Keywords:** Exercise, eHealth, Telemedicine

## Abstract

**Abstract:**

**Introduction:**

Physical inactivity is considered a major public health burden and remains highly prevalent across Gulf Cooperation Council (GCC) countries despite the rapid digital transformation and investment in health information infrastructure. Although technology-based interventions have emerged as promising approaches to promote physical activity, their implementation and impact are still unclear. This scoping review will map technology-based interventions that promote physical activity in the GCC against the domains of the Consolidated Framework for Implementation Research (CFIR) to provide a better understanding of the contexts, implementation mechanisms and outcomes.

**Methods and analysis:**

This review will follow the Joanna Briggs Institute (JBI) methodology for scoping reviews to identify peer-reviewed and grey literature on interventions promoting physical activity delivered through digital technologies among people residing in GCC countries. Six electronic databases (MEDLINE, Embase, CINAHL Plus, Web of Science, Scopus and Epistemonikos), Google Scholar and GCC government websites will be searched. Eligible sources will include technology-based interventions that promote, monitor or track physical activity (eg, mobile apps, SMS, wearables, eHealth/mHealth services, telehealth or web-based platforms). Two reviewers will independently screen titles, abstracts and full texts. Data on study characteristics, technologies, intervention components, outcomes and settings will be charted. A qualitative synthesis of implementation factors will be conducted and mapped to CFIR domains. The review will support future research and policy development in the GCC region.

**Ethics and dissemination:**

Ethical approval is not required as this study involves secondary analysis of published and publicly available data. Findings will be disseminated through peer-reviewed publication and conference presentations.

**STRENGTHS AND LIMITATIONS OF THIS STUDY**

- The review uses a comprehensive search strategy to ensure broad coverage of published and unpublished evidence across multiple databases and grey literature sources with site-specific queries.
- Searches in both English and Arabic will be conducted to enhance capture of regionally relevant interventions.
- The review follows the Joanna Briggs Institute’s guidance and Preferred Reporting Items for Systematic Review and Meta-Analysis extension for Scoping Reviews reporting guidelines to improve rigour and transparency.
- A potential limitation is that government website searches may not capture unpublished or internal reports not housed on official websites.

## Introduction

### Physical inactivity as a global and regional public health issue

 Physical inactivity is a leading global risk factor for morbidity and mortality, contributing substantially to non-communicable diseases (NCDs) such as cardiovascular disease, type 2 diabetes, dementia and certain cancers. According to the World Health Organisation’s Global Status Report on Physical Activity 2022, approximately 31% of adults worldwide, nearly 1.8 billion individuals, do not achieve the minimum recommended levels of physical activity (ie, at least 150 min of moderate-intensity activity or 75 min of vigorous-intensity activity per week for adults and an average of 60 min per day of moderate-to-vigorous activity for children and adolescents). This figure has risen by over five percentage points since 2010.^1 2^ If inactivity rates remain unchanged, an estimated 499 million new NCD cases are expected to emerge by 2030, resulting in direct healthcare costs of approximately US$520 billion globally.^3^

Inactivity rates remain persistently high, particularly in the Middle East and the Gulf Cooperation Council (GCC) region. The prevalence of physical inactivity among adults is estimated at 66% in Saudi Arabia, 62.6% in Kuwait, 45.9% in Qatar and 43.2% in the UAE.^4 5^ In Saudi Arabia, national survey data indicate that only 17% of adults meet WHO-recommended thresholds, leaving 83% insufficiently active.^4 6^ This situation has been further exacerbated by the recent global COVID-19 pandemic, which heightened levels of physical inactivity and sedentary behaviours.^7^ In the broader GCC region, less than one-third of adults in Saudi Arabia and Oman meet recommended levels, compared with a global average above 31%.^1 5^ These rates underscore the urgent need for effective interventions and policy measures to reduce physical inactivity.^8 9^

### Interventions to address physical inactivity

The WHO set a Global Action Plan on Physical Activity (GAPPA) 2018–2030,^9^ which includes a global target of a 15% relative reduction in physical inactivity by 2030^1^ and recommends 20 evidence-based policy actions across sectors.^10^ These include creating active societies through awareness and education campaigns, fostering active environments by designing supportive urban and transport infrastructure, developing active people through school-based and community-based programmes and establishing active systems via stronger governance, partnerships and monitoring mechanisms.^10^ National media campaigns and policy initiatives further support these efforts by shaping norms and raising awareness about the importance of physical activity.^10^ Collectively, these approaches demonstrate that physical activity promotion requires a combination of behavioural, environmental and systemic strategies to achieve sustained impact.^10^

In response to the GAPPA, several GCC countries have incorporated physical activity promotion into their national policy frameworks, such as Saudi Arabia’s Vision 2030^10^ and the UAE’s National Wellbeing Strategy 2031.^11^ These nations share common cultural, social and environmental obstacles to physical activity, including automobile-oriented infrastructure, increased sedentary employment, severe climatic conditions and limited access to active transportation and recreational opportunities^6^. In these countries, cultural and contextual factors play an important role in shaping physical activity behaviours. For example, in Saudi Arabia, where general practitioners play a central role in society, physicians may be able to provide physical activity counselling, yet a lack of skills and time to do so may hinder practice.^12^

Some evidence syntheses report overall positive effects for community-delivered interventions,^13^ including the GCC countries.^14^ For example, Nash and colleagues^14^ identified 14 studies, the majority conducted in Qatar, Saudi Arabia and Oman. The authors report that the studies were highly heterogeneous in terms of design, population and focus, with multicomponent interventions that included dietary behaviours and physical activity.^14^ Another review looking at school-based interventions in the Eastern Mediterranean region^15^ recognised the role of contextual factors, such as culture, climate and environmental barriers to physical activity in this part of the world.^15^ Climate constraints, gender norms affecting public exercise, varying levels of digital literacy and reliance on migrant workforces for most household chores reduce people’s ability and opportunity to engage in various forms of physical activity.^6 16 17^ For instance, women in some GCC countries face additional barriers to outdoor physical activity due to climate and sociocultural expectations, making home-based, technology-based interventions particularly attractive, as some qualitative studies suggest.^18^

### Technology-based interventions to address physical inactivity

Technologies such as smartphone apps, SMS messaging and wearable fitness devices can influence physical activity by prompting people to walk, stand or engage in low-intensity and moderate-intensity activities. Some recent reviews and meta-analyses have reported positive effects, as noted, for example, in Singh *et al*,^19^ or Lee and Park.^20^ The umbrella review of interventions conducted among older adults identified five reviews reporting short-term improvements in physical activity associated with mobile app use.^13^ However, the effects seem to vary across populations, interventions and contextual factors. For instance, a recent umbrella review of digital interventions to promote physical activity and nutrition in young people found limited evidence of their impact on physical activity, due to the heterogeneity of populations, technologies used and the contextual complexity of interventions.^21^ Others revealed differential effects depending on income and socioeconomic status.^22^

Technology-enabled physical activity interventions are particularly relevant in the GCC, where rapid digital transformation of infrastructure and government services is underway. For example, the Sehhaty platform offers a population-level digital health service embedded within Saudi Vision 2030; furthermore, the Quality of Life Programme provides nationwide access to health information and features that can support physical-activity monitoring and promotion.^23^ Similarly, the UAE’s Digital Government Strategy 2025 and Centennial Vision 2071 emphasise the need to include technology-based interventions that promote health and well-being.^11^ In line with this vision, the country has established the Dubai Fitness Challenge, an annual mass-participation initiative that encourages residents to complete 30 min of daily activity for 30 days and leverages digital tools (apps, social media, wearable integration) to boost engagement.^11^ Together, these examples illustrate how GCC governments can deploy digital health infrastructures and time-bound public campaigns to address physical inactivity.

Existing evidence syntheses that include technology-based interventions in both the wider Middle East and North Africa (MENA) region^24^ and GCC countries^14^ showed overall positive effects on physical activity, although effects vary according to the populations and technologies analysed. For example, Nash and colleagues^14^ reported positive effects of pedometer-based interventions on step-count as a measure of physical activity.^14^ Similarly, Tong *et al*’s 2024 review identified 27 mobile-based interventions, of which 7 randomised controlled trials (RCTs) of moderate quality achieved relatively small effect sizes (standardised mean difference=0.42).^24^

Despite this evidence, the heterogeneity of effects across populations, technologies and study designs limits clear conclusions about why some interventions succeed while others do not. Reviews focus primarily on effectiveness outcomes,^14 24^ with limited attention to how interventions were designed, implemented, adapted and sustained within diverse sociocultural and health-system contexts. Tong *et al* review^24^ combines evidence from countries with different political and economic characteristics, including many studies from Iran and Saudi Arabia, followed by Jordan, Oman, Egypt, Kuwait, Lebanon, Qatar and the UAE. The review by Nash *et al*,^14^ although focused on the GCC, included 14 studies, half of which were from Qatar, followed by Saudi Arabia, Oman, Kuwait and the UAE, with studies from Bahrain not reported.^14^ Moreover, this review reported only 11 pedometer-based interventions, mostly derived from the ‘Step into Health’ national programme in Qatar,^14^ making it difficult to compare to other countries and contexts. Furthermore, these reviews relied on evidence coming from peer-reviewed literature.^14 24^ Many technology-based physical activity initiatives in GCC countries may be disseminated outside traditional academic channels, such as government reports, and theses and dissertations often contain substantial documentation of technology-related physical activity initiatives.

The differential effect sizes observed across populations and countries, intervention components and technologies used and outcomes raise the question of what makes technology-based physical activity interventions effective. It is unclear how intervention characteristics, settings and population characteristics interplay and how intervention implementation affects the outcomes. There is also a need to better understand the theoretical frameworks that inform the design of these interventions and how theory translates into behavioural impact.

Implementation science^25^ offers useful frameworks for understanding how and why health interventions succeed or fail beyond their efficacy. Among these, the Consolidated Framework for Implementation Research (CFIR),^26^ developed by Damschroder and colleagues, can serve as an analytical lens to identify factors that influence implementation in complex interventions, such as technology-based physical activity interventions, across multiple levels. CFIR synthesises key constructs from diverse implementation theories into five major domains: intervention characteristics, outer setting, inner setting, characteristics of individuals and implementation process.^26^ These domains capture the complexity of real-world implementation by recognising the influence of organisational, sociocultural, technological and policy contexts, as well as the dynamic processes through which interventions are introduced, adapted and sustained. Applying the CFIR is particularly valuable for technology-based physical activity interventions, which often require alignment among digital features, user capabilities, organisational capacity and broader contextual factors. Currently available reviews of technology-based physical activity interventions in the GCC countries^14 24^ do not provide sufficient information on implementation determinants.

Given the rapid growth of digital health technologies alongside the high burden of physical inactivity in GCC countries, there is a need to move beyond questions of effectiveness and systematically examine how technology-based physical activity interventions are designed, implemented and sustained in real-world contexts. An implementation science-informed synthesis can help elucidate how contextual, organisational and sociocultural factors shape intervention delivery and outcomes, while also highlighting gaps in the use and reporting of theory and implementation frameworks.

### Aims and objectives

To address this need, this scoping review aims to map the existing research on technology-based interventions to promote physical activity in GCC countries, adopting an implementation science perspective. Specifically, the proposed review will: (1) identify the types of technologies and intervention modalities used to promote physical activity across different populations and settings; (2) describe how these interventions are designed, implemented, adapted and evaluated, including delivery agents and settings; and (3) synthesise reported implementation challenges, facilitators and contextual determinants, mapped where possible to domains of the CFIR, influencing reach, adoption, implementation and sustainability.

## Methods

### Methodological frameworks

This scoping review will be conducted using Arksey and O’Malley’s five-step process,^27^ which consists of: (1) identifying the research question; (2) identifying relevant evidence; (3) selecting studies; (4) charting data; and (5) collating, summarising and reporting the results.

The review will follow the reporting guidelines offered by the Joanna Briggs Institute (JBI)’s^28^ and the Preferred Reporting Items for Systematic Reviews and Meta-Analyses extension for Scoping Reviews (PRISMA-ScR)^29^ (see the PRISMA-ScR checklist in online supplemental file 1). The stages of the Arksey and O’Malley framework are described further in the following sections. The review protocol and plan will commence in June 2026. The screening and selection processes will be piloted prior to full screening. The final searches, study selection, data extraction, and analyses will be completed by December 2026.

### Stage 1: identifying the research question

Given the nature of the research objectives, the overarching research question for this scoping review is: How have technology-based interventions to promote physical activity been designed, implemented and evaluated across populations and settings in GCC countries, and what implementation-related factors and frameworks have been reported?

Specific sub-questions include:

1. What types of technologies and intervention modalities have been used to promote physical activity in GCC countries and among which populations and settings?
2. How are these interventions designed, delivered, adapted and evaluated, including delivery agents, implementation strategies and settings?
  What implementation challenges, facilitators and contextual factors are reported as influencing reach, adoption, fidelity and sustainability?
3. What behavioural, theoretical and implementation frameworks are used to inform intervention design and evaluation?

### Stage 2: identifying relevant studies

#### Search strategy

A comprehensive search strategy was developed using the Population-Concept-Context (PCC) framework developed by the JBI for scoping reviews.^28^ We developed three sets of Medical Subject Headings (MeSH) and keywords to define three search concepts as technology-based interventions promoting physical activity (Concept), among people residing in GCC countries (Population/Context).

The search strategy covers both controlled vocabulary (eg, MeSH in MEDLINE and Emtree in Embase) and free-text phrases to capture variations in terminology related to each of the PCC framework components, namely the six GCC nations (Saudi Arabia, the United Arab Emirates, Kuwait, Qatar, Oman, Bahrain), digital technologies (eg, apps, wearables, eHealth, mHealth, digital, telehealth) and physical activity. Boolean and proximity operators (eg, adj3, NEAR/3) were employed as necessary to enhance specificity within each concept. No limitations are placed on study design or publication date; publications in English and Arabic are considered.

A search strategy was initially tested in MEDLINE (Ovid) and later modified for use across other databases to ensure comprehensive, relevant literature coverage. Searches were also piloted in Embase, Scopus, CINAHL Plus and Epistemonikos between April, May and June 2025 to refine search terms and ensure retrieval of relevant studies.

Considering the need to include grey literature and unpublished reports, we will also search official GCC governmental and institutional websites, including ministries of health, public health authorities, digital government agencies and telecommunications regulators. Customised Google search strings will be developed for each website using combinations of physical activity and technology keywords, with Boolean operators and file-type restrictions (eg, (“physical activity” OR exercise OR “physical fitness” OR sports) AND (telemedicine OR mobile OR technology OR digital OR wearable OR smartphone OR eHealth OR mHealth OR “mobile apps” OR “mobile applications”) site:moh.gov.sa file:pdf).

Search results will be exported to Zotero for reference management and deduplication and then imported into Covidence for screening and study selection. The complete search strategies for all databases are provided in online supplemental Appendix A.

#### Information sources

This review will search for evidence from original research articles and review articles that address our research questions. The following databases will be searched: (1) MEDLINE (Ovid), (2) Embase (Ovid) and (3) CINAHL Plus. We will also search Scopus, Web of Science (Core Collection), and Epistemonikos platforms to capture relevant grey literature. The reference lists of included reviews and reports will also be scanned for additional documents.

In addition to peer-reviewed literature, this scoping review will intentionally include grey literature, such as theses/dissertations, government reports, strategic plans, and any other documents that describe the use of technology to promote physical activity in GCC countries.

### Stage 3: study selection

#### Eligibility criteria

The unit of analysis for this review is the intervention or ‘study’ rather than the individual publication describing it. In line with JBI’s PCC framework^28^, we will include technology-based interventions targeting residents of the GCC countries, as defined below.

Population (P): Human participants of any age residing in the six GCC countries (Saudi Arabia, the United Arab Emirates, Bahrain, Kuwait, Qatar and Oman). This review focuses specifically on GCC countries rather than the broader MENA region, because GCC countries share a high-income status, advanced digital health ecosystems, comparable cultural norms, governance structure, health and education policies through the Gulf Health Council^30^ and some of the highest prevalence of NCDs due to behavioural risk factors, including poor diet and physical inactivity.^31^ This relative homogeneity makes the GCC particularly suitable for cross-examining implementation processes. Studies targeting healthy individuals and those living with chronic conditions or rehabilitation (eg, diabetes, cardiovascular disease, obesity) will be included, reflecting the widespread relevance of physical inactivity and the transferability of lessons across population groups.

Concept: Technology-based intervention designed to promote physical activity. In the literature, related terms such as ‘digital interventions’, ‘technology-enabled’ or ‘technology-assisted’ interventions are sometimes used (see studies by Western *et al* and Levine *et al*, for example^22 32^). For consistency, we adopt ‘technology-based interventions’ as an umbrella term encompassing a wide range of digital tools and technology-integrated approaches used in public health and physical activity promotion.

We define technology-based physical activity interventions as any organised public health programme, initiative, campaign or policy-linked action that integrates technology to promote or measure physical activity in GCC countries. This definition draws on the typology of public health interventions proposed by Litvak *et al*,^33^ who describe interventions as ‘an organised set of means implemented in a specific context to meet one or several targets with respect to improving health and preventing disease’. Technology-based interventions may take different forms, including individual-level programmes, community-level or population-level initiatives and policy-linked actions, provided they incorporate a technological component.

Context: Any setting, including community, school, workplace, clinical, or home as long as the intervention is based in GCC countries.

Types of documents: We will consider any document that describes or evaluates a technology-based intervention to promote or measure physical activity that has been conducted in GCC countries. We will consider any source type primary studies (observational, feasibility, usability, development, qualitative, mixed methods), peer-reviewed secondary studies (systematic reviews, scoping reviews, narrative reviews used for contextual purposes) and grey literature (eg, academic papers, government policy documents, non-governmental organisation reports, strategy documents, implementation guidelines) published in any format (eg, full reports, policy briefs, programme evaluations, white papers), in either English or Arabic.

This scoping review will exclude: (1) documents reporting interventions conducted outside GCC countries, (2) documents reporting interventions that do not include technology components (eg, only printed materials, face-to-face counselling without technology) and (3) documents that describe interventions not focused on physical activity. Reasons for exclusion will be documented according to the PCC framework.

#### Study selection process

All references will be managed in Covidence. Screening will occur in two stages: (1) title/abstract and (2) full-text assessment. Each stage will be conducted independently by two reviewers (ASAA and MD). Inclusion and exclusion criteria will be applied in the full-text screening phase and reasons for exclusion will be documented. Prior to formal screening, reviewers will pilot-screen a sample of 20 records to calibrate the application of eligibility criteria.

All disagreements will be discussed; if consensus is not reached, a third reviewer (MB or KE) will adjudicate. For Arabic-language documents, ASAA and KE, both native speakers, will screen them independently. Disagreements will be reviewed by a third reviewer (MB). In addition, regular online meetings will be held with the full review team to discuss any issues arising during the study-selection process.

The number of records at each phase and reasons for exclusion at full text will be documented and presented in a PRISMA-ScR flow diagram.

### Stage 4: charting the data

We will develop and pilot a standardised data-charting form (with three to five studies) and refine as necessary. Given that the unit of analysis is a technology-based physical activity intervention, multiple documents might describe the same intervention. Each intervention will be assigned a numeric ID and information will be charted collectively under a single intervention record.

We will extract information about the document type (eg, peer-reviewed article, thesis, government report), study design (eg, RCT, non-randomised, observational, qualitative, mixed methods) and reported outcomes (physical activity-related, behavioural, anthropometric/clinical, digital health or system-level) with related findings.

To support systematic extraction of implementation-related information, the CFIR^26^ will be used as an organising framework during data charting. Data items will be extracted and mapped to CFIR domains where relevant, recognising that not all domains may be reported for every intervention. The CFIR has been employed to map the implementation of telehealth services^34^ or school mental health programmes.^35^ Data charted will include the main CFIR domains as follows:

- **Intervention identification and characteristics** (*CFIR: Intervention Characteristics):* Intervention ID, intervention name or description, country, technology type (eg, mobile applications, SMS, wearable devices, digital platforms, telehealth, exergaming, emerging technologies), role of technology (core intervention, support tool or measurement only) and intervention components (delivery mode, frequency, duration).
- **Target population and individual-level factors** (*CFIR: Characteristics of Individuals):* Target population characteristics (eg, age group, gender, health status), intended users and any reported user engagement, acceptability or adoption factors.
- **Implementation setting and organisational context** (*CFIR: Inner Setting):* Delivery setting (eg, community, school, workplace, healthcare, home-based, digital), organisational context, infrastructure and implementation supports where reported.
- **Policy and external context** (*CFIR: Outer Setting):* Alignment with national health or digital strategies (eg, Vision 2030 or other national programmes), policy involvement or broader system-level influences.
- **Implementation processes** (*CFIR: Process):* Information on intervention planning, rollout, delivery, monitoring, scale-up, maintenance or adaptation, where available.

### Stage 5: collating, summarising and reporting the results

A narrative synthesis will be used to summarise study characteristics and implementation features of included interventions. Results will be organised and reported using the main CFIR domains as overarching themes and subthemes. Tables and charts will be used to present findings visually (eg, frequency of technology types, target populations).

This mapping will be presented visually (in tables, charts or matrices), which may also inform the development of an Evidence and Gap Map in future work. Such visualisation will help policymakers and stakeholders quickly identify areas with strong evidence and those requiring further research or investment.

#### Critical appraisal

Consistent with methodological guidance for scoping reviews,^28^ a formal critical appraisal or risk-of-bias assessment of individual sources will not be performed in this review. The purpose of the review is to map the breadth, nature and characteristics of available evidence on technology-based physical activity interventions in GCC countries, rather than to evaluate the quality or effectiveness of individual studies. Information on study characteristics, intervention types, and source types will be reported descriptively to support the interpretation of the evidence landscape.

## Strengths and limitations

A strength of this scoping review is that it will follow a rigorous, systematic process to identify, review and summarise evidence addressing our predefined research question. We will follow the steps indicated by Arksey and O’Malley^27^ and document all decisions made at each stage. To enhance reproducibility and transparency, we will follow the existing JBI’s guidance on conducting scoping reviews,^28^ and report the results according to PRISMA-ScR guidelines^29^. Another major strength is that it will adopt an implementation science perspective, informed by the CFIR^26^ to systematically map how technology-based physical activity interventions are designed, implemented and evaluated in GCC countries. Another strength is the inclusion of both peer-reviewed and grey literature, unlike existing evidence syntheses that focus on intervention effectiveness.^14 24^ This review will provide a comprehensive overview of interventions across diverse populations and settings, capturing real-world implementation experiences that are often underreported.

However, as a scoping review, this study will not assess the methodological quality or effectiveness of included studies, and conclusions about intervention impact cannot be drawn. Reporting on implementation determinants and theoretical frameworks is also expected to be variable and may rely on implicit rather than explicitly stated constructs, potentially limiting CFIR mapping. In addition, relevant initiatives may be missed due to language restrictions or limited documentation, particularly in rapidly evolving digital health contexts.

The review will inform future policy and intervention development in GCC countries by systematically identifying and characterising technology-based approaches that have been implemented, mapping them against national health and digital transformation strategies (eg, Saudi Vision 2030, UAE’s National Wellbeing Strategy 2031), and highlighting both context-specific barriers/facilitators and priority gaps in evidence.

## Ethics and dissemination

As the review involves only analysis of publicly available data, ethical approval is not required. The findings will be submitted for publication in relevant academic journals and shared at relevant academic and policy forums.

## Supplementary material

10.1136/bmjopen-2025-110795 online supplemental file 1
