# Supplementary material for: Technology-based interventions to promote physical activity in Gulf Cooperation Council (GCC) countries: a scoping review protocol
Source: BMJ Open. 2026 Jul 20;16(7):e110795. doi: 10.1136/bmjopen-2025-110795 (PMC13386082; doi:10.1136/bmjopen-2025-110795)
Supplement: online supplemental file 1 [file bmjopen-16-7-s001.docx]

Appendix A. Search strategies by database and grey literature sources

**Ovid MEDLINE (1946 to 10 Apr 2025)**
1 saudi arabia/ or united arab emirates/ or kuwait/ or bahrain/ or qatar/ or oman/
2 (Saudi* or Emirat* or Kuwait* or Bahraini* or Qatar* or Qatari* or Oman*).tw.
3 1 or 2
4 exp Exercise/ or exp Physical Fitness/ or exp Motor Activity/ or exp Sports/ or exp Physical Exertion/
5 (physical adj3 (activit* or fit* or train* or exer* or movement)).tw.
6 ((moderate or vigorous) adj activit*).tw.
7 4 or 5 or 6
8 telemedicine/ or mobile applications/ or cell phone/ or wearable electronic devices/ or internet/ or text messaging/ or remote consultation/
9 (technology* or digital* or mobile* or app* or smartphone* or wearable* or eHealth or mHealth or SMS or "short message" or web-based or online or telehealth or telemedicine).tw.
10 8 or 9
11 3 and 7 and 10

**Embase (1974 to 16 Apr 2025)**
1 exp Saudi Arabia/ or exp United Arab Emirates/ or exp Kuwait/ or exp Bahrain/ or exp Qatar/ or exp Oman/
2 (Saudi* or Emirat* or Kuwait* or Bahraini* or Qatar* or Qatari* or Oman*).ti,ab.
3 1 or 2
4 exp Physical Activity/ or exp Exercise/ or exp Fitness/ or exp Motor Activity/ or exp Sports/
5 (physical adj3 (activit* or fit* or train* or exer* or movement)).ti,ab.
6 ((moderate or vigorous) adj3 activit*).ti,ab.
7 4 or 5 or 6
8 exp Mobile Application/ or exp Telemedicine/ or exp Internet/ or exp Text Messaging/
9 (technology* or digital* or mobile* or app* or smartphone* or wearable* or eHealth or mHealth or SMS or "short message" or web-based or online or telehealth or telemedicine).ti,ab.
10 8 or 9
11 3 and 7 and 10

**Scopus (Apr 2025)**
TITLE-ABS-KEY(("Saudi Arabia" OR "Gulf Cooperation Council" OR "United Arab Emirates" OR Kuwait OR Bahrain OR Qatar OR Oman) AND ("physical activity" OR exercise OR "motor activity" OR fitness) AND (technology OR digital OR mobile OR app OR apps OR SMS OR eHealth OR mHealth OR wearable OR telehealth OR "web-based"))

**CINAHL Plus (EBSCO, Apr 2025)**
TX (Saudi* OR Emirat* OR Kuwait* OR Bahraini* OR Qatar* OR Qatari* OR Oman*) AND TX (physical activity OR exercise OR fitness OR "motor activity") AND TX (technology OR digital OR mobile OR app OR apps OR SMS OR eHealth OR mHealth OR wearable OR telehealth OR "web-based")

**Epistemonikos (Apr 2025)**
(title:(("Saudi Arabia" OR "GCC" OR "United Arab Emirates" OR Kuwait OR Bahrain OR Qatar OR Oman) AND ("physical activity" OR exercise OR "motor activity" OR fitness) AND (technology OR mobile OR app OR apps OR SMS OR eHealth OR mHealth OR wearable OR telehealth OR "web-based"))) OR (abstract:(same terms))

**Web of Science (Core Collection, 28 May 2025)**
TS=("Saudi Arabia" OR "United Arab Emirates" OR "UAE" OR Kuwait OR Qatar OR Oman OR Bahrain OR "Gulf Cooperation Council" OR GCC) AND TS=("physical activity" OR exercise OR "motor activity" OR "fitness training") AND TS=(technology OR digital OR mobile OR app OR wearable OR eHealth OR mHealth OR telehealth OR telemedicine OR SMS OR "health application")

**Google Scholar (27 May 2025)**
"physical activity" AND ("technology-based intervention" OR app OR wearable OR eHealth OR mHealth OR digital OR telehealth) AND ("Saudi Arabia" OR "United Arab Emirates" OR "Kuwait" OR "Qatar" OR "Oman" OR "Bahrain" OR "GCC")
Note: we will export the first ~150 records (≈10–15 pages) for screening, as per standard practice.

**Grey literature: government and agency websites (GCC)**
Example site query:
("physical activity" OR exercise OR "physical fitness" OR sports) AND (telemedicine OR mobile OR technology OR digital OR wearable OR smartphone OR eHealth OR mHealth OR "mobile apps" OR "mobile applications") site:[website domain] file:pdf

**Saudi Arabia:** Ministry of Health (MOH); Saudi Food and Drug Authority (SFDA); Public Health Authority (وقاية); National Health Information Center (NHIC); Saudi Data and Artificial Intelligence Authority (SDAIA); Digital Government Authority (DGA); Ministry of Communications and Information Technology (MCIT); Ministry of Human Resources and Social Development (HRSD).

**United Arab Emirates:** Dubai Health Authority (DHA); Ministry of Health and Prevention (MOHAP); Ministry of Education (MOE); Office of the Minister of State for Artificial Intelligence, Digital Economy & Remote Work Applications; Federal Authority for Identity, Citizenship, Customs & Port Security (ICP); Telecommunications and Digital Government Regulatory Authority (TDRA).

**Qatar:** Tasmu Digital Valley (TDV); Qatar Computer Emergency Response Team (Q-CERT); Planning and Statistics Authority – General Statistics Sector (PSA/GSS).

**Kuwait:** Kuwait Direct Investment Promotion Authority (KDIPA); Kuwait Public Policy Center (KPPC); The Supreme Council for Planning and Development (SCPD).

**Bahrain:** Ministry of Health; National Health Information System / Ministry Health Informatics Portal (Health.gov.bh); Ministry of Industry and Commerce (MOIC); Information and eGovernment Authority (IGA).

**Oman:** Ministry of Health; Ministry of Education; State Audit Institution (SAI).

Appendix B. **Preferred Reporting Items for Systematic reviews and Meta-Analyses extension for Scoping Reviews (PRISMA-ScR) Checklist**

| **SECTION** | **ITEM** | **PRISMA-ScR CHECKLIST ITEM** | **REPORTED ON PAGE #** |
| --- | --- | --- | --- |
| **TITLE** | | | |
| Title | 1 | Identify the report as a scoping review. | 1 |
| **ABSTRACT** | | | |
| Structured summary | 2 | Provide a structured summary that includes (as applicable): background, objectives, eligibility criteria, sources of evidence, charting methods, results, and conclusions that relate to the review questions and objectives. | 1, 2 |
| **INTRODUCTION** | | | |
| Rationale | 3 | Describe the rationale for the review in the context of what is already known. Explain why the review questions/objectives lend themselves to a scoping review approach. | 4, 5 |
| Objectives | 4 | Provide an explicit statement of the questions and objectives being addressed with reference to their key elements (e.g., population or participants, concepts, and context) or other relevant key elements used to conceptualize the review questions and/or objectives. | 5 |
| **METHODS** | | | |
| Protocol and registration | 5 | Indicate whether a review protocol exists; state if and where it can be accessed (e.g., a Web address); and if available, provide registration information, including the registration number. | - |
| Eligibility criteria | 6 | Specify characteristics of the sources of evidence used as eligibility criteria (e.g., years considered, language, and publication status), and provide a rationale. | 6, 7 |
| Information sources* | 7 | Describe all information sources in the search (e.g., databases with dates of coverage and contact with authors to identify additional sources), as well as the date the most recent search was executed. | 7, 8 |
| Search | 8 | Present the full electronic search strategy for at least 1 database, including any limits used, such that it could be repeated. | 12, 13, 14 |
| Selection of sources of evidence† | 9 | State the process for selecting sources of evidence (i.e., screening and eligibility) included in the scoping review. | 9 |
| Data charting process‡ | 10 | Describe the methods of charting data from the included sources of evidence (e.g., calibrated forms or forms that have been tested by the team before their use, and whether data charting was done independently or in duplicate) and any processes for obtaining and confirming data from investigators. | 8, 9, 10 |
| Data items | 11 | List and define all variables for which data were sought and any assumptions and simplifications made. | - |
| Critical appraisal of individual sources of evidence§ | 12 | If done, provide a rationale for conducting a critical appraisal of included sources of evidence; describe the methods used and how this information was used in any data synthesis (if appropriate). | - |
| Synthesis of results | 13 | Describe the methods of handling and summarizing the data that were charted. | 8 |
| **RESULTS** | | | |
| Selection of sources of evidence | 14 | Give numbers of sources of evidence screened, assessed for eligibility, and included in the review, with reasons for exclusions at each stage, ideally using a flow diagram. | - |
| Characteristics of sources of evidence | 15 | For each source of evidence, present characteristics for which data were charted and provide the citations. | - |
| Critical appraisal within sources of evidence | 16 | If done, present data on critical appraisal of included sources of evidence (see item 12). | - |
| Results of individual sources of evidence | 17 | For each included source of evidence, present the relevant data that were charted that relate to the review questions and objectives. | - |
| Synthesis of results | 18 | Summarize and/or present the charting results as they relate to the review questions and objectives. | - |
| **DISCUSSION** | | | |
| Summary of evidence | 19 | Summarize the main results (including an overview of concepts, themes, and types of evidence available), link to the review questions and objectives, and consider the relevance to key groups. | - |
| Limitations | 20 | Discuss the limitations of the scoping review process. | - |
| Conclusions | 21 | Provide a general interpretation of the results with respect to the review questions and objectives, as well as potential implications and/or next steps. | - |
| **FUNDING** | | | |
| Funding | 22 | Describe sources of funding for the included sources of evidence, as well as sources of funding for the scoping review. Describe the role of the funders of the scoping review. | 10 |

JBI = Joanna Briggs Institute; PRISMA-ScR = Preferred Reporting Items for Systematic reviews and Meta-Analyses extension for Scoping Reviews.

* Where *sources of evidence* (see second footnote) are compiled from, such as bibliographic databases, social media platforms, and Web sites.

† A more inclusive/heterogeneous term used to account for the different types of evidence or data sources (e.g., quantitative and/or qualitative research, expert opinion, and policy documents) that may be eligible in a scoping review as opposed to only studies. This is not to be confused with *information sources* (see first footnote).

‡ The frameworks by Arksey and O’Malley (6) and Levac and colleagues (7) and the JBI guidance (4, 5) refer to the process of data extraction in a scoping review as data charting*.*

§ The process of systematically examining research evidence to assess its validity, results, and relevance before using it to inform a decision. This term is used for items 12 and 19 instead of "risk of bias" (which is more applicable to systematic reviews of interventions) to include and acknowledge the various sources of evidence that may be used in a scoping review (e.g., quantitative and/or qualitative research, expert opinion, and policy document).

*From:* Tricco AC, Lillie E, Zarin W, O'Brien KK, Colquhoun H, Levac D, et al. PRISMA Extension for Scoping Reviews (PRISMAScR): Checklist and Explanation. Ann Intern Med. 2018;169:467–473. [doi: 10.7326/M18-0850](http://annals.org/aim/fullarticle/2700389/prisma-extension-scoping-reviews-prisma-scr-checklist-explanation).
