# Supplementary material for: Technology-based interventions to promote physical activity in Gulf Cooperation Council (GCC) countries: a scoping review protocol
Source: BMJ Open. 2026 Jul 20;16(7):e110795. doi: 10.1136/bmjopen-2025-110795 (PMC13386082; doi:10.1136/bmjopen-2025-110795)
Supplement: Supplementary file 2 [file bmjopen-2025-110795.reviewer_comments.pdf]

PEER REVIEW HISTORY

BMJ Open publishes all reviews undertaken for accepted manuscripts. Reviewers are asked to complete a checklist review form and are provided with free text boxes to elaborate on their assessment. These free text comments are reproduced below.

ARTICLE DETAILS

Title (Provisional)

Technology Based Interventions to Promote Physical Activity in Gulf Cooperation Council (GCC) Countries: A Scoping Review Protocol

Authors

Alghamdi, Asma Saad A; Bardus, Marco; Das, Manami; Elmusharaf, Khalifa

---

VERSION 1 - REVIEW

**Reviewer**	**1**
**Name**	**Spijker, Jeroen**
**Affiliation**	**Universitat Internacional de Catalunya, IESF**
**Date**	**23-Mar-2026**
**COI**	**None**

The protocol addresses an important and regionally relevant question. The Gulf Cooperation Council (GCC) countries have very high levels of physical inactivity, and a review that maps technology-enabled approaches in their specific policy and sociocultural contexts could be useful. The manuscript also has some strengths: it aims to search both peer-reviewed and grey literature, proposes English and Arabic searches, and states that it will follow PRISMA-ScR and JBI guidance. However, in its current form, I think the protocol needs clearer justification of novelty and a more explicit scoping-review methodology before it is suitable for publication.

My main concern is overlap with prior reviews, especially for the “direct actions” category. The authors’ proposed direct actions include mobile apps, SMS, wearables, telehealth counselling, and web-based platforms. This substantially overlaps with Tong et al. 2024, which already systematically reviewed mobile technologies for physical activity and sedentary behaviour in the MENA region, including apps, fitness trackers, and SMS, and explicitly discussed cultural fit, user perspectives, and implementation-related issues. Tong et al. also notes the earlier Nash et al. 2021 GCC review as part of the existing evidence base.

Indeed, overlap of the proposed scoping review also exists with Nash et al. 2021, which systematically reviewed physical activity interventions in the six GCC countries regardless of delivery mode and included pedometer-based programmes, smartphone app/pedometer

interventions, text-message reminders, workplace prompts, counselling, and multicomponent approaches. Their review already covered several interventions that the present protocol seems likely to retrieve under “direct actions,” including Step into Health studies in Qatar and app- or message-supported interventions in Oman, Qatar, Saudi Arabia, Kuwait, and the UAE.

For that reason, the authors should sharpen the rationale and explicitly explain what this scoping review adds beyond those two reviews. At present, the added value appears to be the inclusion of broader evidence types beyond evaluative intervention studies, namely advocacy campaigns, support interventions, and policy/strategic initiatives. That could be a legitimate contribution, but it needs to be stated much more clearly. As written, the protocol risks looking like a partial update or repackaging of prior systematic reviews for one major segment of the evidence base, while adding several much broader categories that are insufficiently defined.

I would therefore recommend that the authors do one of two things. Either they narrow the review question so that it focuses on the areas not already well covered by earlier reviews, such as policy, strategy, campaigns, implementation ecosystems, and support infrastructures for technology-enabled physical activity promotion in GCC countries; or they retain the broad scope but clearly structure the review as a mapping exercise across distinct evidence domains, while explicitly acknowledging that “direct actions” will overlap with prior systematic reviews and are included here mainly to contextualise the wider ecosystem. Without that clarification, the contribution is not yet sufficiently distinct.

A second major issue is methodological clarity. BMJ Open scoping review protocols usually lay out the scoping-review framework more explicitly, see e.g. Cheng et al. (BMJ Open 2020;10:e038876. doi:10.1136/bmjopen-2020-038876) who state state that their protocol is based on Arksey and O’Malley, refined by JBI, and then explicitly list the six stages of a scoping review. They also provide a table of inclusion and exclusion criteria organised by Population, Concept, and Context, which makes the protocol much easier to understand and assess. Osman et al. (BMJ Open 2018;8:e022733. doi:10.1136/bmjopen-2018-022733) similarly specify the Arksey and O’Malley/Levac/JBI basis, define the research question, present a structured selection-criteria table, explain why certain related models are excluded, and discuss the optional consultation stage.

By contrast, while this manuscript refers to the PCC framework, PRISMA-ScR, and JBI, it does not yet provide enough detail on how the scoping review will actually be conducted as a scoping review rather than simply as a broad literature search. I suggest the authors explicitly add a short subsection describing the methodological framework, for example: identification of the research question; identification of relevant sources; study selection; data charting; collating, summarising and reporting results; and optional stakeholder consultation. This would align the paper more closely with BMJ Open scoping-review protocol norms.

Relatedly, the eligibility criteria need to be much more transparent. At present, the categories of “direct actions,” “advocacy campaigns,” “support interventions,” and “collaborative or policy/strategic initiatives” are interesting, but they are too broad and potentially heterogeneous.

The authors also need to be clearer about the unit of analysis. They say they will include “interventions, campaigns, strategies, or population-level policies.” These are not equivalent units. Some are studies, some are programmes, some are policy documents. That is acceptable in a scoping review, but only if the review questions and charting framework are built around that heterogeneity. Otherwise, the synthesis may become conceptually muddled.

A further point concerns quality appraisal. Scoping reviews generally do not require formal risk-of-bias assessment, and BMJ Open protocols often state this explicitly. Osman does so directly, while still noting that basic study characteristics and source types will be reported. The current manuscript should be explicit on whether critical appraisal will be performed. If not, that should be stated and justified.

Finally, the study-selection procedure is insufficiently specified. The protocol states that two reviewers will independently screen studies, but it does not explain how disagreements will be resolved. This is a notable omission. In comparable protocols, disagreement resolution is stated explicitly. Cheng et al. indicate that disagreements are discussed first by the two reviewers and then adjudicated by a third reviewer if needed. Osman et al. similarly note that unresolved disagreements are reconciled by a supervisor, while Spijker et al. (2024; https://www.researchprotocols.org/2024/1/e59900) state that each paper is reviewed by at least two people, with conflicts resolved by a third person, and that team meetings are held to address issues arising during study selection. Given the breadth and heterogeneity of the present review, this should be specified clearly for both title/abstract screening and full-text review. Moreover, while two independent reviewers may be sufficient in principle for study selection in a scoping review, this protocol covers a particularly broad and heterogeneous evidence base, including peer-reviewed studies, grey literature, government websites, policy documents, campaigns, and interventions across six countries and two languages, meaning that the feasibility, consistency, and reproducibility of the selection process cannot be assumed and should be supported by a clearly prespecified conflict-resolution procedure, reviewer calibration, and clarification of whether additional team members will assist with grey-literature searching, Arabic-language screening, and data charting.

VERSION 1 - AUTHOR RESPONSE

Response to Reviewer 1

We thank the reviewer for the very constructive feedback. We have carefully addressed each point raised and revised the protocol accordingly. Below, we provide a point-by-point response outlining the revisions made to the manuscript and clarifying our rationale. A tracked-changes version of the manuscript has been uploaded.

Reviewer Comment 1.1

The protocol addresses an important and regionally relevant question. The Gulf Cooperation Council (GCC) countries have very high levels of physical inactivity, and a review that maps technology-enabled approaches in their specific policy and sociocultural contexts could be useful. The manuscript also has some strengths: it aims to search both peer-reviewed and grey literature, proposes English and Arabic searches, and states that it will follow PRISMA-ScR and JBI guidance. However, in its current form, I think the protocol needs clearer justification of novelty and a more explicit scoping-review methodology before it is suitable for publication.

Response 1.1

Thank you for this positive and constructive assessment. We appreciate the reviewer's suggestion to strengthen both the justification of novelty and the clarity of the scoping review methodology.

In response, we have substantially revised the Abstract, Introduction, and Methods sections to more clearly articulate the unique contribution of this review and to align the methodology with established scoping review guidance.

Specifically:

- We clarified that the review addresses an implementation-focused research question, rather than effectiveness.
- We strengthened the rationale and positioning of the review in relation to existing evidence.
- We revised the Methods section to explicitly describe the scoping review framework, including the stages of the review and the application of the PCC (Population–Concept–Context) approach.

These revisions are reflected in the updated manuscript (Introduction and Methods sections).

Reviewer Comment 1.2:

My main concern is overlap with prior reviews, especially for the "direct actions" category. The authors' proposed direct actions include mobile apps, SMS, wearables, telehealth counselling, and web-based platforms. This substantially overlaps with Tong et al. 2024, which already systematically reviewed mobile technologies for physical activity and sedentary behaviour in the MENA region, including apps, fitness trackers, and SMS, and explicitly discussed cultural fit, user perspectives, and implementation-related issues. Tong et al. also notes the earlier Nash et al. 2021 GCC review as part of the existing evidence base.

Indeed, overlap of the proposed scoping review also exists with Nash et al. 2021, which systematically reviewed physical activity interventions in the six GCC countries regardless of delivery mode and included pedometer-based programmes, smartphone app/pedometer interventions, text-message reminders, workplace prompts, counselling, and multicomponent approaches. Their review already covered several interventions that the present protocol seems likely to retrieve under "direct actions," including Step into Health studies in Qatar and app- or message-supported interventions in Oman, Qatar, Saudi Arabia, Kuwait, and the UAE.

For that reason, the authors should sharpen the rationale and explicitly explain what this scoping review adds beyond those two reviews. At present, the added value appears to be the inclusion of broader evidence types beyond evaluative intervention studies, namely advocacy campaigns, support interventions, and policy/strategic initiatives. That could be a legitimate contribution, but it needs to be stated much more clearly. As written, the protocol risks looking like a partial update or repackaging of prior systematic reviews for one major segment of the evidence base, while adding several much broader categories that are insufficiently defined.

I would therefore recommend that the authors do one of two things. Either they narrow the review question so that it focuses on the areas not already well covered by earlier reviews, such as policy, strategy, campaigns, implementation ecosystems, and support infrastructures for technology-enabled physical activity promotion in GCC countries; or they retain the broad scope but clearly structure the review as a mapping exercise across distinct evidence domains, while explicitly acknowledging that "direct actions" will overlap with prior systematic reviews and are included here mainly to contextualise the wider ecosystem. Without that clarification, the contribution is not yet sufficiently distinct.

Response 1.2:

Thank you for this important and insightful comment. We agree that the distinction between the present review and prior evidence syntheses needs to be clearly articulated. In response, we have revised the Introduction and Rationale sections to explicitly position this review in relation to existing studies, particularly Tong et al. (2024) and Nash et al. (2021).

Our review differs from these studies in several key respects:

First, in terms of research focus:
Existing reviews primarily examine the effectiveness of physical activity interventions, including digital approaches. In contrast, our review addresses an implementation-focused question, examining how technology-based interventions are designed, delivered, adapted, and sustained in real-world GCC contexts, and what factors influence these processes.

Second, in terms of geographic scope**:**
We intentionally focus on GCC countries, rather than the broader MENA region. While MENA-wide reviews are valuable, they include substantial heterogeneity in health systems, socioeconomic contexts, and digital infrastructure. The relative homogeneity of GCC countries enhances the relevance of comparative implementation insights and strengthens policy applicability.

Third, in terms of scope of technology and evidence**:**
Unlike prior reviews that focus primarily on mobile technologies or peer-reviewed intervention studies, this review includes a broader range of technology-based interventions (e.g., wearable, telehealth, web-based, and system-level digital initiatives) and incorporates both peer-reviewed and grey literature, including government programmes and policy-linked initiatives.

Fourth, in terms of analytical approach**:**
This review adopts an implementation science lens, using the Consolidated Framework for Implementation Research (CFIR) to systematically map implementation determinants across interventions. This enables a structured analysis of contextual, organisational, and process-related factors that are not captured in prior effectiveness-focused reviews.

We acknowledge that some individual-level digital interventions included in this review may overlap with those identified in previous syntheses. However, these are included to contextualise the broader implementation ecosystem, rather than to reassess intervention effectiveness. This revised positioning is now explicitly reflected in the Introduction (pages 6–7), where we provide a critical synthesis of existing evidence from Tong et al. (2024) and Nash et al. (2021) to clarify the gap addressed by this review.

Reviewer Comment 1.3:

A second major issue is methodological clarity. BMJ Open scoping review protocols usually lay out the scoping-review framework more explicitly, see e.g. Cheng et al. (BMJ Open 2020;10:e038876. doi:10.1136/bmjopen-2020-038876) who state state that their protocol is based on Arksey and O'Malley, refined by JBI, and then explicitly list the six stages of a scoping review. They also provide a table of inclusion and exclusion criteria organised by Population, Concept, and Context, which makes the protocol much easier to understand and assess. Osman et al. (BMJ Open 2018;8:e022733. doi:10.1136/bmjopen-2018-022733) similarly specify the Arksey and O'Malley/Levac/JBI basis, define the research question, present a structured selection-criteria table, explain why certain related models are excluded, and discuss the optional consultation stage.

By contrast, while this manuscript refers to the PCC framework, PRISMA-ScR, and JBI, it does not yet provide enough detail on how the scoping review will actually be conducted as a scoping review rather than simply as a broad literature search. I suggest the authors explicitly add a short subsection describing the methodological framework, for example: identification of the research question; identification of relevant sources; study selection; data charting; collating, summarising and reporting results; and optional stakeholder consultation. This would align the paper more closely with BMJ Open scoping-review protocol norms.

Response 1.3:

Thank you for this important methodological suggestion. We agree that the scoping review framework needed to be more explicitly described.

In response, we have revised the Methods section to clearly outline the methodological approach underpinning this review. Specifically, we now state that the review is guided by Arksey and O'Malley's framework, with refinements from the Joanna Briggs Institute (JBI) guidance for scoping reviews.

We have explicitly described the five stages of the scoping review process, including:

1. Identifying the research question
2. Identifying relevant studies
3. Study selection
4. Data charting
5. Collating, summarising, and reporting the results

In addition, we clarified the application of the Population–Concept–Context (PCC) framework in defining the research question, search strategy, and eligibility criteria.

A formal stakeholder consultation stage will not be conducted; however, the findings will be interpreted with relevance to policy and implementation stakeholders.

These revisions are reflected in the Methods section (pages 8–13), where we explicitly describe the five stages of the scoping review, the application of the PCC framework in the search strategy and eligibility criteria, and the documentation of reasons for exclusion.

Reviewer Comment 1.4:

Relatedly, the eligibility criteria need to be much more transparent. At present, the categories of "direct actions," "advocacy campaigns," "support interventions," and "collaborative or policy/strategic initiatives" are interesting, but they are too broad and potentially heterogeneous.

The authors also need to be clearer about the unit of analysis. They say they will include "interventions, campaigns, strategies, or population-level policies." These are not equivalent

units. Some are studies, some are programmes, some are policy documents. That is acceptable in a scoping review, but only if the review questions and charting framework are built around that heterogeneity. Otherwise, the synthesis may become conceptually muddled.

Response 1.4:

Thank you for this important comment. We agree that greater clarity was needed regarding the eligibility criteria and the unit of analysis.

In response, we have made the following revisions:

First, unit of analysis:
We now explicitly define the unit of analysis as the technology-based intervention, rather than the individual publication. We clarify that multiple documents (e.g., peer-reviewed articles, reports, or policy documents) describing the same intervention will be grouped and analysed as a single intervention record.

Second, eligibility criteria:
We have simplified and clarified the eligibility criteria by consistently using the term "technology-based interventions", removing previously used categories (e.g., "direct actions," "advocacy campaigns," "support interventions") that could introduce ambiguity. Campaigns, strategies, and policies are included where they meet the definition of a technology-based intervention.

Third, handling heterogeneity:
Rather than restricting inclusion, we now explicitly structure heterogeneity through key classification dimensions, including intervention level (individual, organisational, population), technology type, target population, and setting.

Fourth, analytical framework:
To ensure a coherent synthesis across diverse intervention types, we use the Consolidated Framework for Implementation Research (CFIR) to systematically organise and interpret implementation-related data.

This approach aligns with public health intervention frameworks, where programmes, campaigns, and policies can be conceptualised as population-level interventions when they are designed to influence health behaviours.

These revisions are reflected in the Methods section, where we explicitly define the intervention as the unit of analysis, clarify the handling of multiple documents describing the same intervention, and specify the use of the CFIR framework to structure data charting and synthesis.

Reviewer Comment 1.5:

A further point concerns quality appraisal. Scoping reviews generally do not require formal risk-of-bias assessment, and BMJ Open protocols often state this explicitly. Osman does so directly, while still noting that basic study characteristics and source types will be reported. The current manuscript should be explicit on whether a critical appraisal will be performed. If not, that should be stated and justified.

Response 1.5:

Thank you for this helpful comment. We agree that this needed to be stated explicitly. In response, we have clarified in the Methods section that no formal critical appraisal or risk-of-bias assessment will be conducted, in line with methodological guidance for scoping reviews.

We emphasise that the purpose of this review is to map the breadth, nature, and characteristics of the available evidence, rather than to evaluate the methodological quality or effectiveness of individual studies.

These revisions are now clearly stated in the manuscript (Methods section).

Reviewer Comment 1.6:

Finally, the study-selection procedure is insufficiently specified. The protocol states that two reviewers will independently screen studies, but it does not explain how disagreements will be resolved. This is a notable omission. In comparable protocols, disagreement resolution is stated explicitly. Cheng et al. indicate that disagreements are discussed first by the two reviewers and then adjudicated by a third reviewer if needed. Osman et al. similarly note that unresolved disagreements are reconciled by a supervisor, while Spijker et al. (2024; https://www.researchprotocols.org/2024/1/e59900) state that each paper is reviewed by at least two people, with conflicts resolved by a third person, and that team meetings are held to address issues arising during study selection. Given the breadth and heterogeneity of the present review, this should be specified clearly for both title/abstract screening and full-text review. Moreover, while two independent reviewers may be sufficient in principle for study selection in a scoping review, this protocol covers a particularly broad and heterogeneous evidence base, including peer-reviewed studies, grey literature, government websites, policy documents, campaigns, and interventions across six countries and two languages, meaning that the feasibility, consistency, and reproducibility of the selection process cannot be assumed and should be supported by a clearly prespecified conflict-resolution procedure, reviewer calibration, and clarification of whether additional team members will assist with grey-literature searching, Arabic-language screening, and data charting.

Response 1.6:

Thank you for this important comment. We agree that the study selection process and procedures for resolving disagreements required further clarification.

In response, we have expanded the Study Selection Process section in the Methods to provide a clear and structured description.

Specifically:

- Screening will be conducted in two stages (title/abstract and full-text), with two independent reviewers at each stage.
- A pilot screening exercise will be conducted to ensure consistency in applying eligibility criteria.
- Disagreements will be resolved through discussion between reviewers, and, where necessary, adjudication by a third reviewer.
- For Arabic-language documents, screening will be conducted independently by two native-speaking reviewers, with disagreements resolved by a third reviewer.
- In addition, regular team meetings will be held to address any uncertainties and ensure consistency throughout the screening process.

The full study selection procedure, including the use of Covidence, pilot screening, and detailed conflict-resolution processes, is now explicitly described in the Methods section. These revisions improve the transparency and reproducibility of the study selection process
